# Biological Nitrogen Removal through Nitritation Coupled with Thiosulfate-Driven Denitritation

**DOI:** 10.1038/srep27502

**Published:** 2016-06-08

**Authors:** Jin Qian, Junmei Zhou, Zhen Zhang, Rulong Liu, Qilin Wang

**Affiliations:** 1 School of Architecture and Civil Engineering, Chengdu University, Sichuan 610106, China; 2 Department of Civil and Environmental Engineering, The Hong Kong University of Science and Technology, Clear Water Bay, Kowloon, Hong Kong 00852, China; 3 State Key Laboratory of Heavy Oil Processing, Beijing Key Laboratory of Oil and Gas Pollution Control, China University of Petroleum, Beijing 102249, China; 4 Advanced Water Management Centre (AWMC), The University of Queensland, QLD 4072, Brisbane, Australia

## Abstract

A novel biological nitrogen removal system based on nitritation coupled with thiosulfate-driven denitritation (Nitritation-TDD) was developed to achieve a high nitrogen removal rate and low sludge production. A nitritation sequential batch reactor (nitritation SBR) and an anoxic up-flow sludge bed (AnUSB) reactor were applied for effective nitritation and denitritation, respectively. Above 75% nitrite was accumulated in the nitritation SBR with an influent ammonia loading rate of 0.43 kg N/d/m^3^. During Nitritation-TDD operation, particle sizes (d_50_) of the sludge decreased from 406 to 225 um in nitritation SBR and from 327–183 um in AnUSB reactor. Pyrosequencing tests revealed that ammonium-oxidizing bacteria (AOB) population was stabilized at approximately 7.0% (calculated as population of AOB-related genus divided by the total microbial population) in the nitritation SBR. In contrast, nitrite-oxidizing bacteria (NOB) population decreased from 6.5–0.6% over the same time, indicating the effective nitrite accumulation in the nitritation SBR. *Thiobacillus*, accounting for 34.2% in the AnUSB reactor, was mainly responsible for nitrogen removal via autotrophic denitritation, using an external source of thiosulfate as electron donor. Also, it was found that free nitrous acid could directly affect the denitritation activity.

Due to the effectiveness, economics and environmental friendliness, biological nitrogen removal (BNR) process has been widely used to remove wastewater nutrient (e.g. nitrogen) in order to protect lakes and other natural water against eutrophication^1^. The traditional BNR process mainly relies on the combined nitrification (i.e. NH_4_^+^ → NO_2_^−^ → NO_3_^−^) with heterotrophic denitrification (i.e. NO_3_^−^* → *N_2_). However, nitrification requires intensive aeration energy consumption and heterotrophic denitrification is usually restricted by the insufficient organic carbon^2^. Compared to the traditional nitrification-heterotrophic denitrification (i.e. NH_4_^+^ → NO_2_^−^ → NO_3_^−^* → *N_2_), the combination of nitritation (i.e. NH_4_^+^ → NO_2_^−^) with heterotrophic denitritation (i.e. NO_2_^−^ → N_2_) not only reduces the oxygen consumption by 25%, but also saves the organic carbon requirement by 40%^3^. Additional advantages include the higher nitrogen removal rate and lower sludge production^4^,^5^. However, carbon source is still required in the heterotrophic denitritation.

Autotrophic denitrification is becoming more economically attractive in the last two decades, with its advantages including: (I) no external organic carbon source consumption; and (II) much less sludge production^6^,^7^. Since thiosulfate (S_2_O_3_^2−^) dependent autotrophic denitrification was reported in both anaerobic sludge^8^ and pure culture^9^, several studies have been conducted on this new sulfur source-based BNR process through both batch and continuous tests^10^,^11^,^12^,^13^. For instance, it was found that thiosulfate could induce a much higher denitrification activity in terms of both nitrogen removal and sulfate generation rates compared with elemental sulfur and sulfide induced denitrification^14^,^15^. Also, through the batch test, Chung *et al.*^16^ found that the biomass yield in thiosulfate-based denitritation was 36% less than that in thiosulfate-based denitrification.

In this study, a novel process for effective biological nitrogen removal was developed coupling nitritation (dissolved oxygen (DO) was 2.2~2.5 mg/L; temperature was around 25 °C) with thiosulfate-driven denitritation (Nitritation-TDD) (see Fig. 1). The optimal pH for nitritation was determined through batch tests and then applied to the nitritation reactor. The reactor performance and sludge property of this nitritation coupled with thiosulfate-driven denitritation (Nitritation-TDD) process was evaluated. 454 pyrosequencing was carried out to assess the microbial community shift during the operation of this Nitritation-TDD process. As free nitrous acid (FNA, i.e. HNO_2_) would affect heterotrophic denitrification/denitritation activity^17^,^18^,^19^, the effect of FNA on thiosulfate-based autotrophic denitritation activity was also investigated.

## Results and Discussion

### Batch Test I: effect of pH on nitritation

Effective nitrite accumulation in the nitrification reactor could be influenced by a lot of factors, such as DO, pH, temperature, salinity and so on^20^. Among these factors, DO and pH were more widely investigated and could significantly affect nitrite accumulation in biological nitrification reactor^21^. Also, since DO and pH are already able to achieve a high nitrite accumulation ratio (>75%, see the following section), only these two factors were considered in this study. DO was one of the dominant factors for nitritation process and low DO (under 1.5 mg/L) could achieve 75% nitrite accumulation by retaining ammonia oxidizing bacteria (AOB) while eliminating nitrite oxidizing bacteria (NOB)^17^. However, N_2_O, which is a well-know greenhouse gas and has a very strong global warming potential, could be accumulated significantly under low DO condition^22^. Tallec *et al.*^23^ found that N_2_O emission rate became significantly higher when the DO concentration decreased to below 2.0 mg/L. Meanwhile, the nitrite accumulation through the low DO level in the reactor would be at the cost of the decrease in ammonia oxidizing rate^21^. Therefore, DO was controlled at above 2.0 mg/L in this study and pH effect on nitritation was studied.

The ammonium and nitrite profiles under different pH, but the same DO condition (2.2~2.5 mg/L) and under the temperature of 25 ^o^C, are shown in Fig. 2. Below pH 6.0 and 7.0, ammonia oxidizing and nitrite accumulating rates were much lower (p < 0.05) than those under pH 8.0 and 9.0. Therefore, the AOB activity was depressed to a large extent when pH decreased from 8.0 to 7.0. Specifically, ammonia oxidizing rate was reduced by 45% when pH decreased from 8.0 to 7.0 (calculated from Fig. 2a). Although AOB activity was still high under pH 9.0, ammonia was more inclined to be converted to NO_3_^−^ instead of NO_2_^−^ compared to that under pH 8.0 (see Fig. 2b). These results showed that the nitritation activity (NH_4_^+^ → NO_2_^−^) at pH 8.0 was similar (p > 0.05) to that at pH 9.0. But the nitratation activity (NO_2_^−^* → *NO_3_^−^) at pH 9.0 was much faster (p < 0.05) than that at pH 8.0, leading to the quick conversion of NO_2_^−^ to NO_3_^−^. Consequently, pH 9.0 is unsuitable for nitrite accumulation and pH 8.0 with the biomass-specific ammonia removal rate of 48.4 mg NH_4_^+^-N/g MLVSS/h (calculated from Fig. 2a) was selected for the nitritation sequential batch reactor (nitritation SBR).

pH has a direct and indirect (via FNA and free ammonia) effect on the activities of AOB and NOB. During the batch test, the average FNA concentrations in Batch Reactors A (pH = 6.0), B (pH = 7.0), C (pH = 8.0) and D (pH = 9.0) were 3.8 × 10^−2^, 3.8 × 10^−3^, 3.8 × 10^−4^ and 2.5 × 10^−5^ mg HNO_2_-N/L (calculated from Fig. 2b and Eq. 1), respectively. According to the literatures^24^,^25^, the inhibition on AOB and NOB initiated at an FNA concentration of 0.10 and 0.066 mg HNO_2_-N/L, respectively, which were much higher than the FNA concentrations in our study. Therefore, FNA would not inhibit activities of AOB and NOB in our study and thus did not play a role in the washout of NOB. The average free ammonia (FA) concentration in Batch Reactors A (pH = 6.0), B (pH = 7.0), C (pH = 8.0) and D (pH = 9.0) were around 0.017, 0.17, 1.7 and 10 mg NH_3_-N/L (calculated from Fig. 2a and Eq. 2). Based on the literatures^26^,^27^, the inhibition on AOB initiated at an FA concentration of 70 mg NH_3_-N/L and NOB was capable of acclimating to an FA level as high as 22 mg NH_3_-N/L. Therefore, FA would not affect AOB and NOB activities and thus did not contribute to the NOB washout in our study. As a result, the NOB washout in our study was probably due to the direct effect of pH.


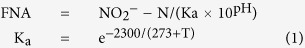


where T = 25 °C in this study


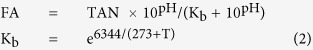


where TAN is total ammonia concentration (NH_4_^+^-N + NH_3_-N), T = 25 °C in this study.

### Nitrogen removal performance in Nitritation-TDD system

Based on the results of the above batch test, pH 8.0 was selected in nitritation SBR for effective nitrite accumulation. Figure 3a shows the ammonia, nitrate and nitrite concentrations during the nitritation SBR operation. From Day 5, the ammonia could be totally removed from nitritation SBR and then its removal efficiency was stabilized at 100% afterwards (see Fig. 3a). Also, with the pH of 8.0 and DO of 2.2~2.5 mg/L, the nitrite accumulation ratio (Effluent NO_2_^−^-N concentration/Influent NH_4_^+^-N concentration × 100%) gradually increased to above 75% with less than 10 mg NO_3_^−^-N/L being detected in the nitritation SBR effluent within one month. Afterwards, the nitrite accumulation ratio maintained at above 75%. This suggested the strong nitritation activity and the possible elimination of NOB in the nitritation SBR, which was confirmed by the following microbial community analysis results.

Interestingly, the total nitrogen (TN) loss percentage ((Influent NH_4_^+^-N–Effluent NH_4_^+^-N–Effluent NO_2_^−^-N–Effluent NO_3_^−^-N)/Influent NH_4_^+^-N) in the nitritation SBR was found to be about 25% at the beginning of the nitritation SBR operation (Fig. 3b). Similar finding was also reported by Luo *et al.*^28^, in which about 30% TN loss was observed from an aerobic SBR. The reason for this TN loss could be attributed to the heterotrophic denitrifying bacteria (i.e. *Thaeura*, see microbial results). The sludge in the nitritation SBR existed in the granule-like form (see the next Section), which led to the existence of anoxic zone in the inner part of the nitritation SBR sludge. This would cause the occurrence of heterotrophic denitrification/denitritation. With the operation of the nitritation SBR, the TN loss percentage gradually decreased and reached below 10% (Fig. 3b) at the end of the operation, corresponding to the decrease of sludge particle size (see the following section) and the disappearance of *Thaeura* genus (Table 1). In addition, a COD removal of above 90% was also observed in the nitritation SBR.

For the denitritation reactor (i.e. anoxic up-flow sludge bed reactor: AnUSB reactor), the denitritation efficiency could reach 100% after 7 days’ operation with thiosulfate as the electron donor (see Fig. 3c). In other words, no nitrate and nitrite were detected in the effluent of the AnUSB reactor. Also, more than 90% of thiosulfate was converted to sulfate (sulfite was not detected) in the AnUSB reactor. Therefore, based on the nitration in the nitritation SBR and thiosulfate-driven denitritation in the AnUSB reactor, influent ammonia with the loading rate of 0.43 kg N/d/m^3^ (influent ammonia concentration of 72 mg N/L and Nitritation-TDD system’s total hydraulic retention time (HRT) of 4 h) could be completely removed in this novel Nitritation-TDD system.

In contrast, in the sulfide-based biological nitrogen removal system including full nitrification and sulfide-driven nitrate denitrification, the nitrogen removal rate was only 0.18 kg N/d/m^29^. Therefore, the nitrogen removal potential could be increased with nitritation coupled with thiosulfate-driven denitritation.

### Particle size distribution in Nitritation-TDD system

During the Nitritation-TDD system operation, particle size (d_50_) in both nitritation SBR and AnUSB reactor decreased gradually (p < 0.05). Initially, particle size (d_50_) of the sludge in the nitritation SBR was 406 um, but decreased to 296 um on Day 41 and then to 225 um (p < 0.05) at the end of the operation. Similarly, particle size (d_50_) dramatically dropped (p < 0.05) from 327 um to 206 um and then to 183 um in the AnUSB reactor. The relatively large particle size indicated that the sludge existed in the granule-like form^30^. The decreased particle size might be due to the fact that the extracellular polymeric substances (EPS) were disintegrated by FNA existing in both reactors^31^−^33^, leading to the generation of smaller particles. Wang *et al.*^33^ speculated that the FNA-induced smaller particles were likely to accumulate to some extent, leading to relatively smaller particle sizes in the sludge. However, the FNA concentration applied in the study of Wang *et al.*^33^ was 2.0 mg HNO_2_-N/L. In contrast, the FNA concentration could only accumulate to 1.42 × 10^−3^ mg HNO_2_-N/L in the Nitritation SBR and to 1.07 × 10^−3^ mg HNO_2_-N/L in the AnUSB reactor. Therefore, the reason for the decreased particle size still needs further study in the future.

### Sludge production in Nitritation-TDD system

The sludge production in both nitritation SBR and AnUSB reactor was calculated and the profile of biomass in terms of MLVSS (MLVSS: mixed liquor volatile suspended solids) was shown in Fig. 4. The NH_4_^+^ removal-specific sludge yield in the nitritation SBR was approximately 0.39 g MLVSS/g NH_4_^+^-N (see Fig. 4a). According to the literature, the pure nitrifying biomass yield was in the range of 0.10–0.17 g MLVSS/g NH_4_^+^-N^34^, which was lower than what we achieved. The reason could be attributed to the heterotrophic biomass growth (at a yield of 0.46 g MLVSS/g COD)^35^ in our system due to the influent COD of 75 mg/L. The NH_4_^+^ removal-specific sludge yield in the nitritation SBR (i.e. 0.39 g MLVSS/g NH_4_^+^-N) was also much lower than that (i.e. 1.60 g MLVSS/g NH_4_^+^-N) achieved in the SBR with an alternate phase (anoxic-aerobic phases)^33^.

In the AnUSB reactor, the nitrogen removal-specific sludge yield was 0.37 g MLVSS/g NO_x_^−^-N (NO_x_^−^-N = NO_2_^−^-N + NO_3_^−^-N) (detailed calculation was shown in Fig. 4b). This value was slightly higher than that of 0.34 mg MLVSS/g NO_2_^−^-N in the study of Chung *et al.*^16^. The difference might be due to the facts that: I) heterotrophic denitrification (based on the solubilized EPS and/or cell lysate) could also contribute to the N removal in our AnUSB reactor; 2) small amount of NO_3_^−^ from the nitritation SBR effluent could enter the AnUSB reactor to enable full denitrification to happen. Therefore, it is reasonable that a higher sludge yield was achieved in the AnUSB reactor. However, this value (0.37 g MLVSS/g NO_x_^−^-N) was still lower than NO_3_^−^ based autotrophic denitrification (average yield of 0.49 g MLVSS/g NO_3_^−^-N^36^) and NO_3_^−^- based heterotrophic denitrification (average yield of 1.0 g MLVSS/g NO_3_^−^-N^37^). Also, this yield was lower than 0.57 g MLVSS/g NO_3_^−^-N in sulfide-based NO_3_^−^ autotrophic denitrification^38^. Overall, the sludge production in this Nitritation-TDD system was quite low.

### Batch Test II: effect of FNA on denitritation activity

In the first four batch reactors in Batch Test II (Batch Reactors 1, 2, 3 and 4), denitritation activities with the same pH level, but different NO_2_^−^ concentrations (i.e. different FNA concentrations, see Table 2) were tested. The biomass-specific denitritation rate was 523 mg NO_2_^−^-N/g MLVSS/d (see Table 2, detailed results of Batch Test II are shown in Fig. S1a) with the lowest NO_2_^−^ level (i.e. 30 mg NO_2_^−^-N/L). When the initial NO_2_^−^ concentration increased to 120 mg NO_2_^−^-N/L, the denitritation activity (91 mg NO_2_^−^-N/g MLVSS/d) in Batch Reactor 4 was only 17% of that in Batch Reactor 1. Therefore, there was a general negative effect of nitrite/FNA on denitritation activities.

In the other four batch reactors (Batch Reactors 5, 6, 7 and 8), effects of pH on denitritation activity were investigated. With the same initial nitrite concentration in each reactor (60 mg NO_2_^−^-N/L), the denitritation rate was highest at pH 9.0 among all the tested pH conditions (Table 2). The nitrogen removal rate decreased sharply with the decreased pH value (detailed batch test results are shown in Fig. S1b). When the denitritation rates were plotted against the FNA concentrations, the correlation was very strong (see Fig. S1c). This indicated that the thiosulfate-driven denitritation activity was directly influenced by FNA (both nitrite and pH) instead of nitrite or pH alone. The detailed mechanism of possible FNA effect on thiosulfate-based denitritation would be revealed in the future study.

In addition, the 8 control tests without thiosulfate were also performed during the Batch Test II. About 10% to 20% of nitrite was consumed in the control reactors in the absence of S_2_O_3_^2−^ (data not shown here). It manifests that besides thiosulfate, other electron donors could also play a role in the nitrogen removal in the AnUSB reactor. One possible explanation was that some organics were released through EPS disintegration when the anoxic sludge was exposed to the FNA^39^. The released organics and/or cell lysate were then utilized by the heterotrophic denitrifying biomass in the AnUSB reactor (see the following microbial results).

### Microbial community analysis

Four sludge samples were collected from the two reactors: nitritation SBR sludge on Day 0 (N0), nitritation SBR sludge on Day 72 (N72), AnUSB sludge on Day 0 (A0) and AnUSB sludge on Day 72 (A72), to analyze the microbial shift in each reactor. The sequence tags were assigned to different operational taxonomic units with a 3% nucleotide cutoff according to the ribosomal database project RDP.

During 72 days’ operation, microbial structure communities in the nitritation SBR reactor changed obviously at the genus level. One reported AOB genus (i.e. *Nitrosomonas*) and two reported NOB genera (i.e. *Nitrospira* and *Nitrobacter*) were detected in the sludge from nitritation SBR. The AOB (*Nitrosomonas*) level at the end of nitritation SBR operation (6.6%, calculated as the population of Nitrosomonas divided by the total amount of microbial communities) was almost the same as that at the initial period (7.4%) (see Table 1). In contrast, the population of NOB related genus (*Nitrospira* and *Nitrobacter*, calculated as the population of *Nitrospira* and *Nitrobacter* divided by the total amount of microbial communities) dropped dramatically, from 2.8% and 3.7% in the beginning, to 0.33% and 0.26% at the end of the operation. Thus, from the microbial point of view, the maintenance of AOB and washout of NOB corresponded to the high nitritation activity and nitrite accumulation in the nitritation SBR (see Fig. 3a).

It is also noteworthy that the *Thauera* was also detected at 5.6% on Day 0 in nitritation SBR, but decreased to 0.52% at the end. *Thauera* was reported to be a normal kind of denitrifying bacteria^40^, which could exist in the nitrifying granular sludge reactor^28^. At the initial stage, the nitritation SBR sludge was in the granule-like form with the particle size (d_50_) of 406 um. Therefore, the anoxic zone could be formed in the nitritation SBR sludge and *Thauera* could be cultivated. After 72 days, the particle size was reduced to 225 um and sludge was more in the floc-like form with little anoxic part in the sludge. Therefore, the growth of denitrifier in the nitritation SBR would be significantly inhibited.

*Thiobacillus,* affiliated to Betaproteobacteria, was the most abundant genus (34.2%) in the AnUSB reactor (see Table 1). When coupled with reduced sulfur (sulfide and thiosulfate) oxidation, it could reduce nitrate to nitrogen gas^41^,^42^. In the case of our study, the AnUSB reactor was fed with thiosulfate. So this condition facilitated the enrichment of *Thiobacillus* population, making it the dominant genus in the AnUSB reactor. Its level increased from 23.4–34.2% after 72 days’ operation, suggesting denitrification activity was enhanced, corresponding to the high nitrogen removal rate in the AnUSB reactor (see Fig. 3c). *Sulfurovum* was reported to be a mesophilic genus under Epsilon-proteobacteria class, which could oxidize thiosulfate to sulfate as the end product in the denitrification process for its chemo-lithoautotrophic growth^43^,^44^. They were found at 4.3% at the genus level at the end of AnUSB reactor operation. In addition, *Shinella*, an autotrophic denitrifying genus to reduce NO_2_^−^ to N_2_^44^, was observed at 2.2% in the AnUSB reactor (see Table 1).

Besides the autotrophic denitrifying biomass, a certain level of heterotrophic denitrifier, i.e. *Thauera* (6.4%) was also found in the AnUSB reactor, which could possibly utilize the organics from EPS disintegration and/or cell lysate as the electron donors. The observation of the *Thauera* could explain the nitrite reduction without electron donor (thiosulfate) in the control reactors in Batch Test II (see the results of Batch Test II above) and confirm that heterotrophic denitrification could also play a role in the nitrogen removal in the AnUSB reactor.

### Implications of this work

In this study, we proposed a novel process for effective biological nitrogen removal through nitritation coupled with thiosulfate-driven denitritation. Although thiosulfate is not directly available in the wastewater, our previous study has shown that thiosulfate could be the end product of biological sulfate reduction (organics as the electron donor) under low pH (e.g. pH 6.5) and low temperature (e.g. 15 °C)^45^,^46^. Therefore, large amounts of thiosulfate could be produced from the sulfate containing industrial wastewater or seawater during biological sulfate reduction. The produced thiosulfate would then be available for autotrophic denitritation/denitrification using nitrite/nitrate as the electron acceptor, which was produced in the nitritation/nitrification reactor and then recycled to the autotrophic denitritation/denitrification reactor. Therefore, the thiosulfate supply and the proposed process are feasible for the real application. Also, it should be noted that the thiosulfate driven denitritation rates were in the range of 50~1250 mg NO_2_^−^-N/g MLVSS/d (see Table 2). In contrast, the organics driven heterotrophic denitritation/denitrification rates are generally in the range of 30~110 mg N (NO_2_^−^ or NO_3_^−^)/g MLVSS/d^47^,^48^. These are comparable with or lower than the thiosulfate driven denitritation rates. In addition, the Nitritation-TDD system has a lower sludge production. As a result, the cost for nitrogen removal through the Nitritation-TDD process was supposed to be lower than that via the heterotrophic nitrogen removal. However, this still needs further study and verification in the future. In addition, although removing/separating COD from the wastewater in the beginning (e.g. in high-rate reactor) is the goal of most novel nitrogen removal process (e.g. high-rate reactor followed by autotrophic nitrogen removal), only 60~80% of COD can be removed/separated in the high-rate reactor in practice (personal communication with industry partners). Therefore, COD was added to the nitritation SBR to mimic the “real world” situation in our study.

## Materials and Methods

### Sludge cultivation

#### Nitrifying sludge cultivation

Nitrifying biomass was enriched in a 2.4 L sequential batch reactor (SBR). The return activated sludge from a municipal wastewater treatment plant was used as the inoculum. The sludge was cultivated for more than two months. The biomass concentration in SBR was 3200 mg MLVSS/L while reaching steady state. Dissolved oxygen (DO) was controlled at 2.0~3.0 mg/L. The SBR was operated at 4 hours per cycle with an exchange ratio of 0.5 (HRT = 8 hrs; HRT: hydraulic retention time). The nitrogen and chemical oxygen demand (COD) (glucose was used as COD in this study) concentrations were 240 mg NH_4_^+^-N/L and 480 mg COD/L, respectively, in the SBR influent. 1 L nutrient stock solution (composition was shown in Table S1) was diluted to 20 L by freshwater in the SBR influent. The other conditions of the nitrifying sludge cultivation in SBR were shown in Table S2. After cultivation for two months, ammonia removal efficiency ((Influent NH_4_^+^-N concentration–Effluent NH_4_^+^-N concentration)/Influent NH_4_^+^-N concentration × 100%) reached above 95%.

#### Autotrophic denitrifying sludge cultivation

An anoxic up-flow sludge bed (AnUSB) reactor with an effective volume of 1.2 L was adopted for autotrophic denitrifying sludge cultivation. The raw sludge was taken from an anoxic tank of a wastewater treatment plant. The initial concentration of the seeding sludge was about 5200 mg MLVSS/L. For the sludge cultivation, 120 mg N/L of nitrate was supplied as the electron acceptor while sulfide (120 mg S/L) and thiosulfate (240 mg S/L) as the electron donors (ratio of S_2_O_3_^2−^-S to HS^−^ was 2:1). The nominal HRT was set at 8 hours (the nitrogen loading rate was 0.36 kg N/d/m^3^) and the temperature was controlled at 25 °C. All the other nutrients for the influent of the AnUSB reactor was the same as that used for the nitrifying sludge cultivation (see Table S1). The influent pH of the reactor was adjusted to about 8.0 by HCl. The AnUSB reactor achieved almost 100% nitrate removal without nitrite accumulation under the steady state after two months’ cultivation. The MLVSS concentration was around 2800 mg/L after reaching steady state.

### Batch Test I: determining optimal pH for nitritation

After the nitrifying sludge cultivation was completed in the SBR, Batch Test I was carried out to determine the optimal pH for nitritation before the Nitritation-TDD system operation. The nitrifying sludge was taken out from the SBR and then was washed with distilled water for three times to remove background substrates (i.e. ammonia, nitrite, etc). Afterwards, the nitrifying sludge was equally distributed into four 1 L batch reactors (flask) (i.e. Batch Reactors A, B, C and D). pH in each reactor was controlled at 6.0, 7.0, 8.0 and 9.0, respectively, by Na_2_HPO_4_/NaH_2_PO_4_ buffer solution. 60 mg NH_4_^+^-N/L and 60 mg/L COD of glucose were dosed as the nitrogen and carbon source, respectively. DO was controlled at about 2.2~2.5 mg/L by the air pump in the four reactors. The temperature was kept at 25 ± 1 °C in all reactors in an air-conditioning room. The tests lasted for 6 hours, during which the sludge samples were taken regularly for the analysis of ammonium, nitrite and nitrate. Based on the results of these tests, the optimal pH for ammonia oxidization and nitrite accumulation (i.e. highest ammonia oxidization rate and nitrite accumulation) was determined and then applied to the nitritation SBR of the Nitritation-TDD system.

### Set-up and operation of Nitritation-TDD system for biological nitrogen removal

**Figure 1:**
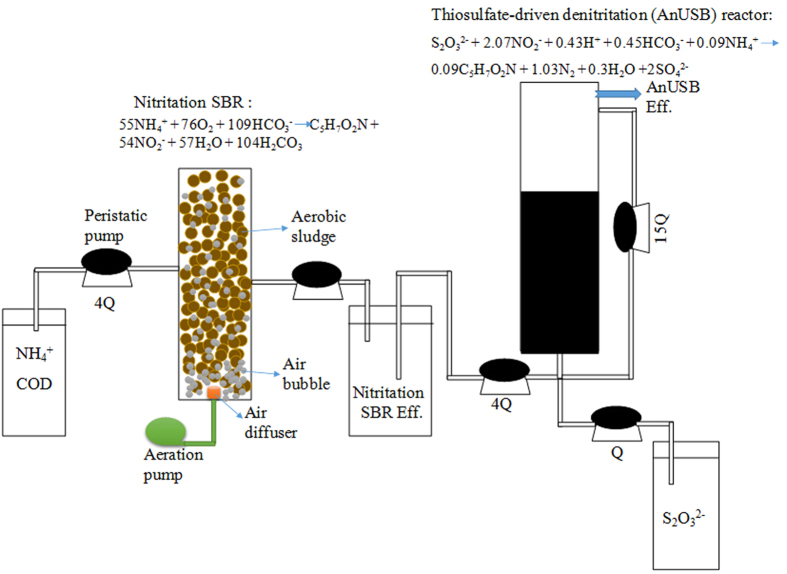
Schematics of the proposed Nitritation-TDD system, including a nitritation (nitritation SBR) and a denitritation (AnUSB) reactor.

**Figure 2:**
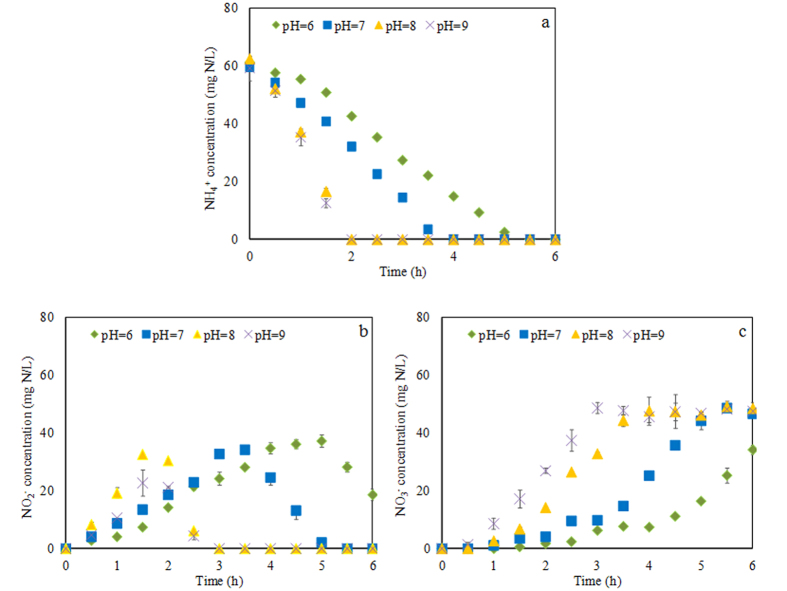
Results of Batch Test I: (**a**) profiles of ammonia, (**b**) nitrite and (**c**) nitrate.

After the successful cultivation of both nitrifying and autotrophic denitrifying sludge, the lab-scale biological nitrogen removal system based on nitritation and thiosulfate-driven denitritation was set up (see Fig. 1). Half of the cultivated nitrifying sludge in the above-mentioned 2.4 L SBR was transferred to a 1 L nitritation SBR for nitritation reaction. Influent ammonia and COD (using glucose) concentrations for the nitritation SBR were about 72 mg NH_4_^+^-N/L and 75 mg COD/L. The molar ratio of bicarbonate (NaHCO_3_) to ammonium-nitrogen in the nitritation SBR influent was set at 4.0 in order to maintain pH at 7.9–8.0^28^, which was chosen based on the Batch Test I results. This was to ensure the highest ammonia oxidization rate and nitrite accumulation. Each cycle for nitritation SBR lasted for 1 hour with an exchange ratio of 0.5 (HRT = 2 h), including 3 min-feeding, 40 min-aeration, 15 min-settling and 2 min-decanting. DO was controlled at 2.2~2.5 mg/L in nitritation SBR. The initial MLVSS concentration of nitritation SBR was 2860 mg/L.

**Figure 3:**
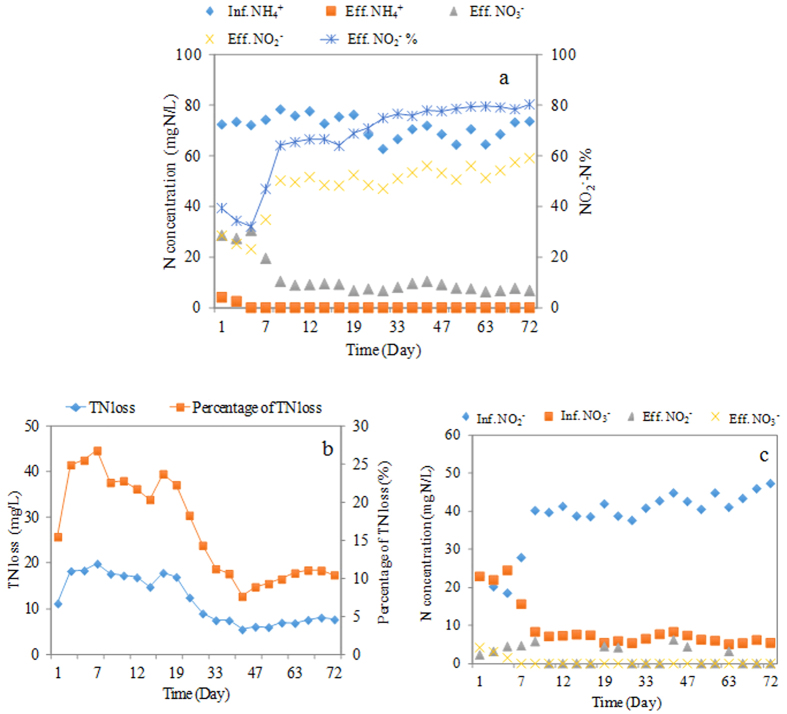
Profiles of (**a**) nitrification performance in nitritation SBR, (**b**) TN loss in nitritation SBR, and (**c**) denitrification performance in AnUSB reactor during Nitritation-TDD system operation. TN loss = Influent NH_4_^+^-N–Effluent NH_4_^+^-N–Effluent NO_2_^−^-N–Effluent NO_3_^−^-N. Percentage of TN loss = (Influent NH_4_^+^-N–Effluent NH_4_^+^-N–Effluent NO_2_^−^-N–Effluent NO_3_^−^-N)/Influent NH_4_^+^-N.

**Figure 4:**
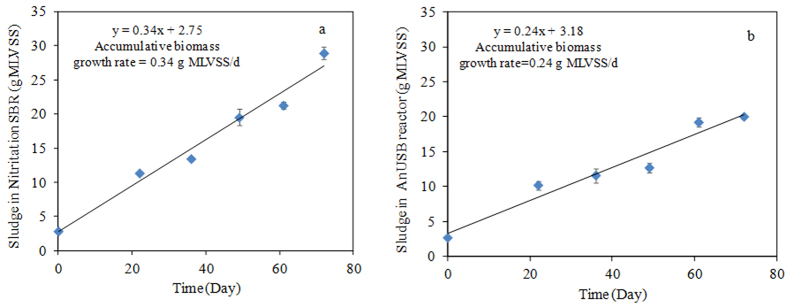
Accumulative biomass in terms of g MLVSS during Nitritation-TDD system operation. (**a**) sludge production in nitritation SBR was calculated as accumulative biomass growth rate (g MLVSS/d) ÷ average ammonia removal rate^a^; (**b**) sludge production in AnUSB reactor was calculated as accumulative biomass growth rate (g MLVSS/d) ÷ average NO_x_^−^ (NO_2_^−^ + NO_3_^−^) removal rate^b^. ^a^Average ammonia removal rate = 72 mg NH_4_^+^-N/L × 1 L × (24 h/day ÷ 2 h_HRT_)/1000 = 0.864 g NH_4_^+^-N/d. ^b^Average NO_x_^−^ (NO_2_^−^ + NO_3_^−^) removal rate = 45.06 mg NO_x_^−^-N/L × 1.2 L × (24 h/day ÷ 2 h_HRT_)/1000 = 0.649 g NO_x_^−^-N/d.

The nitritation SBR was connected with the AnUSB reactor (1.2 L; HRT of 2 h) for Nitritation-TDD system operation. Under such an operating condition, nitrite concentration in nitritation SBR effluent was as high as above 50 mg N/L with less than 10 mg NO_3_^−^-N/L detected (see Fig. 3). Sodium thiosulfate solution at a concentration of 900 mg S_2_O_3_^2−^-S/L was prepared separately to supply S source for the AnUSB reactor for denitritation. For the influent of the AnUSB reactor, the nitritation SBR effluent and sodium thiosulfate solution were pumped to the reactor simultaneously by two peristaltic pumps, with the flow rate of nitritation SBR effluent being 4 times as quick as that of sodium thiosulfate solution (see Fig. 1). So the actual influent S_2_O_3_^2−^ concentration of the AnUSB reactor was 180 mg S/L (i.e. 900/5). According to the mass ratio of S_2_O_3_^2−^-S to NO_2_^−^-N of 2.5 to 1^16^, 72 mg NO_2_^−^-N/L in the AnUSB influent could be totally removed theoretically. pH in the AnUSB reactor was controlled at 7.9–8.0 through the pH controller. In order to ensure the efficient substrate transfer between bulk liquid and biomass in the AnUSB reactor, an internal recycle ratio of 3 (influent flow rate is 5Q, the recycle flow rate is 15Q) was applied (see Fig. 1). The initial MLVSS concentration of AnUSB reactor was 2240 mg/L. The temperature was kept at 25 °C in an air conditioning room.

During the operation of the Nitritation-TDD system, the influent and effluent of both nitritation SBR and AnUSB reactor were sampled regularly for the analysis of total nitrogen (TN), ammonia, nitrite, nitrate, COD and sulfur species (i.e. sulfate, sulfite and thiosulfate). Sludge samples of both nitritation SBR and AnUSB reactor were periodically taken for the analysis of MLVSS concentration and particle size measurement. The structure of microbial communities in both nitritation SBR and AnUSB reactor was analyzed at the beginning (Day 0) and the end (Day 72) of the Nitritation-TDD system operation.

### Batch test II: determining effect of FNA on autotrophic denitritation activity

In order to test the effect of FNA on thiosulfate-based autotrophic denitritation activity in the AnUSB reactor, the autotrophic denitrifying sludge in AnUSB reactor was taken out for batch test at the end of the Nitritation-TDD system operation. According to Eq. 3, FNA concentration depends on NO_2_^−^ concentration, pH level and temperature. In our study, the temperature was kept constant at 25 °C and FNA effect was tested by varying NO_2_^−^ concentration and pH. Therefore, Batch Reactors 1–4 were set up to test the FNA effect by controlling pH at 7.5 via Na_2_HPO_4_/NaH_2_PO_4_ buffer solution, but varying the initial NO_2_^−^ concentration from 30–120 mg N/L (see Table 2). In contrast, Batch Reactors 5–8 were to test the FNA effect by maintaining the same initial NO_2_^−^ concentration at 60 mg N/L, but varying pH level from 6.0–9.0 (see Table 2). Each batch reactor (500 mL flask) was purged with helium gas for half an hour to exclude the oxygen in order to maintain the anaerobic conditions prior to each test. All flasks were sealed with butyl rubber stoppers and aluminum crimp seals and were stirred using a magnetic stirrer at 150 rpm. The MLVSS concentration in each reactor was about 620 mg/L. 360 mg S_2_O_3_^2−^-S/L thiosulfate was dosed into each reactor, which is stoichiometrically sufficient for nitrogen removal in all the reactors. In addition to the above-mentioned 8 batch reactors, another 8 control reactors (Control Reactors 1 to 8) without electron donors (i.e. thiosulfate) were also set up accordingly and operated in parallel (see Table S3). The test lasted for 24 hours. The denitritation activity was determined by the biomass-specific NO_2_^−^ reduction rate, which was calculated from the slope of nitrite concentration, versus time (d), normalized by the biomass concentration in the reactor. It was reported in mg N/g MLVSS/d. The results reported are average values of three replicates. Standard deviation values were under 5%.

### Microbial analysis

#### DNA extraction, PCR amplification and pyrosequencing

Sludge samples from nitritation SBR and AnUSB reactor were collected at the beginning (Day 0) and the end (Day 72) of the Nitritation-TDD system operation to analyze the structure of microbial communities. The samples were collected by centrifugation under 12,000 rpm for 10 minutes. Around 0.5 g of the pellet was weighted for each sample and stored at −80 °C until the DNA extraction. Genomic DNA was extracted using the PowerSoil DNA Isolation Kit (MoBio Laboratories, Inc., Carlsbad, CA) following the manufacturer’s protocols. The quality and quantity of DNA were checked with a NanoDrop device (ND-1000, Thermo Fisher, USA).

The primer pair 515 F and 926 R targeting the V1 and V3 regions was used to amplify the bacterial 16 S rRNA gene^49^. Barcode sequences were incorporated between the 454 adaptor and the forward primer (Table S4). Each of 100 μL PCR reaction mixture contained 5 U of Pfu Turbo DNA polymerase (Stratagene, La Jolla, CA, USA), 1× Pfu reaction buffer, 0.2 μM of dNTPs (TaKaRa, Dalian, China), 0.1 μM of each primer and 20 ng of genomic DNA template. PCR was performed with a thermal cycler (Bio-Rad, USA). The thermal cycles include an initial denaturation at 94 °C for 5 min, followed by 30 cycles of 94 °C for 30 s, 53 °C for 30 s and 72 °C for 45 s; and a final extension at 72 °C for 10 min. The PCR products were purified using Agarose Gel DNA Purification Kit (TaKaRa, China) and quantified with the NanoDrop device. The purified PCR amplicons were sequenced using the ROCHE 454 FLX Titanium platform (Roche, Basel, Switzerland) at the National Human Genome Centre of China (Shang Hai, China).

#### Data analysis

The data analysis followed the procedures reported in Qian *et al.*^50^. Briefly, the raw pyrosequencing reads were firstly subject to quality filtering to remove sequences containing more than one ambiguous ‘N’ or shorter than 150 bps^51^, and to check the completeness of the barcodes and the adaptor. The remained sequences were then aligned and clustered into operational taxonomic units (OTUs) using the MOTHUR program with 97% as the threshold^45^. The representative sequences from each OTU were picked and the taxonomic classifications were assigned using the RDP Classifier^52^. Alpha diversity of each sample was calculated in MOTHUR.

### Chemical analysis

Total organic carbon (TOC) and total nitrogen (TN) was measured by a TOC/TN analyzer (Shimadzu 5000 A). A flow injection analyzer (QuikChem 8500, Lachat Instruments) was applied to measure the concentration of ammonium. Nitrite and nitrate were detected with an ion chromatograph (HIC-20 A super) equipped with a conductivity detector and an IC-SA2 analytical column. Chemical oxygen demand (COD) and mixed liquor volatile suspended solid (MLVSS) was measured according to the Standard Method^53^. Sulfate and thiosulfate were detected by ion chromatography (IC). Sulfite concentration was obtained by titration as detailed in Qian *et al.*^15^. pH and temperature were monitored using a multi-meter electrode during each test (WTW multi 3420). Particle size distribution was assessed using Mastersizer 2000 series (Malvern Instruments, Worcestershire, UK) on the basis of volumetric distribution.

## Additional Information

**How to cite this article**: Qian, J. *et al.* Biological Nitrogen Removal through Nitritation Coupled with Thiosulfate-Driven Denitritation. *Sci. Rep.* **6**, 27502; doi: 10.1038/srep27502 (2016).

## Supplementary Material

Supplementary Information

## Figures and Tables

**Table 1:** Relative abundances of nitrifying and denitrifying-related genera in nitritation SBR and AnUSB reactor.

Sludge samples		Day 0 (%)	Day 72 (%)
Nitritation SBR	*Nitrosomonas (AOB)*	7.4	6.6
	*Nitrospira (NOB)*	2.8	0.33
	*Nitrobacter (NOB)*	3.7	0.26
	*Thauera*	5.6	0.52
AnUSB reactor	*Thiobacillus*	21.8	34.2
	*Sulfurovum*	1.6	4.3
	*Shinella*	0	2.2
	*Thauera*	0.6	6.4

**Table 2:** Results of Batch Test II–biomass-specific denitritation activities under different initial NO_2_^−^ concentrations, pH and FNA concentrations.

Batch reactor	pH	Initial NO_2_^−^ concentration (mg N/L)	Initial FNA concentration (mg N/L)	Biomass-specific NO_2_^−^ reduction rate (mg N/g MLVSS/d)
1	7.5	30	0.002	523
2	7.5	60	0.004	233
3	7.5	90	0.006	143
4	7.5	120	0.008	91
5	6.0	60	1.35 × 10^−1^	51
6	7.0	60	1.35 × 10^−2^	72
7	8.0	60	1.35 × 10^−3^	831
8	9.0	60	1.35 × 10^−4^	1254
