# Supplementary material for: Biological Nitrogen Removal through Nitritation Coupled with Thiosulfate-Driven Denitritation
Source: Sci Rep. 2016 Jun 8;6:27502. doi: 10.1038/srep27502 (PMC4897740; doi:10.1038/srep27502)
Supplement: Supplementary Information [file srep27502-s1.pdf]

Biological Nitrogen Removal through Nitritation Coupled with Thiosulfate-Driven Denitritation

(Supplementary Information)

Jin Qian[1,2], Junmei Zhou[1,2], Zhen Zhang[2,3*], Rulong Liu[2], Qilin Wang[4*]

[1]School of Architecture and Civil Engineering, Chengdu University, Sichuan 610106, China

[2]Department of Civil and Environmental Engineering, The Hong Kong University of Science and Technology, Clear Water Bay, Kowloon, Hong Kong 00852, China

[3]State Key Laboratory of Heavy Oil Processing, Beijing Key Laboratory of Oil and Gas Pollution Control, China University of Petroleum, Beijing 102249, China

[4]Advanced Water Management Centre (AWMC), The University of Queensland, QLD 4072, Brisbane, Australia

Correspondence and requests for materials should be addressed to

Q.W. (email: q.wang@awmc.uq.edu.au) and

Z.Z. (email: zhangzhen_23102@163.com).

Table S1. Composition of stock nutrient solution for both nitrifying sludge[a] and AnUSB reactor.

Component	Concentration (g/L)	Component	Concentration (g/L)
NH_4Cl	18.45	$FeCl_3·6H_2O$	2
K_2HPO_4	1.92	H_3BO_3	0.2
KH_2PO_4	0.72	$CuSO_4$	0.05
$MgCl_2·6H_2O$	8.32	KI	0.08
$CaCl_2$	5.2	$MnSO_4·4H_2O$	0.25
$NaHCO_3$	62.4	$ZnSO_4·7H_2O$	0.15
		$CoCl_2·6H_2O$	0.2

[a]Organic carbon (as glucose) was additionally dosed to the nitrifying sludge, resulting in an influent COD concentration of 480 mg COD/L.

Table S2. Conditions for the nitrifying sludge cultivation.

Effective reactor volume (L)	2.4
Exchange ratio	0.5
Temperature (^oC)	23 ± 1
pH	7.5~8.0
HRT (hrs)	8
DO concentration (mg/L)	2~3
Influent NH_4^+ concentration (mg N/L)	240
Influent COD concentration (mg COD/L)	480
MLVSS concentration (mg/L)	3200

Table S3. Conditions (i.e. pH, initial NO_2^- and $S_2O_3^{2-}$ concentrations) for the 8 batch reactors and 8 control reactors (without $S_2O_3^{2-}$ as the electron donor) in Batch Test II – biomass-specific denitritation activities under different initial NO_2^- concentrations, pH and FNA concentrations.

		pH	NO_2^- conc. (mg N/L)	$S_2O_3^{2-}$ conc. (mg S/L)
Batch Reactors	1	7.5	30	360
	2	7.5	60	
	3	7.5	90	
	4	7.5	120	
	5	6.0	60	
	6	7.0	60	
	7	8.0	60	
	8	9.0	60	
Control Reactors	1	7.5	30	0
	2	7.5	60	
	3	7.5	90	
	4	7.5	120	
	5	6.0	60	
	6	7.0	60	
	7	8.0	60	
	8	9.0	60	

Table S4. Primer of the DNA amplification for NSBR and AnUSB reactor at the beginning and end of operation.

Barcode Sequence	Primer
ATGCTACGTC	(V1-V3）<br>8F: 5’-AGAGTTTGATCCTGGCTCAG-3’<br>533R: 5’-TTACCGCGGCTGCTGGCAC-3’

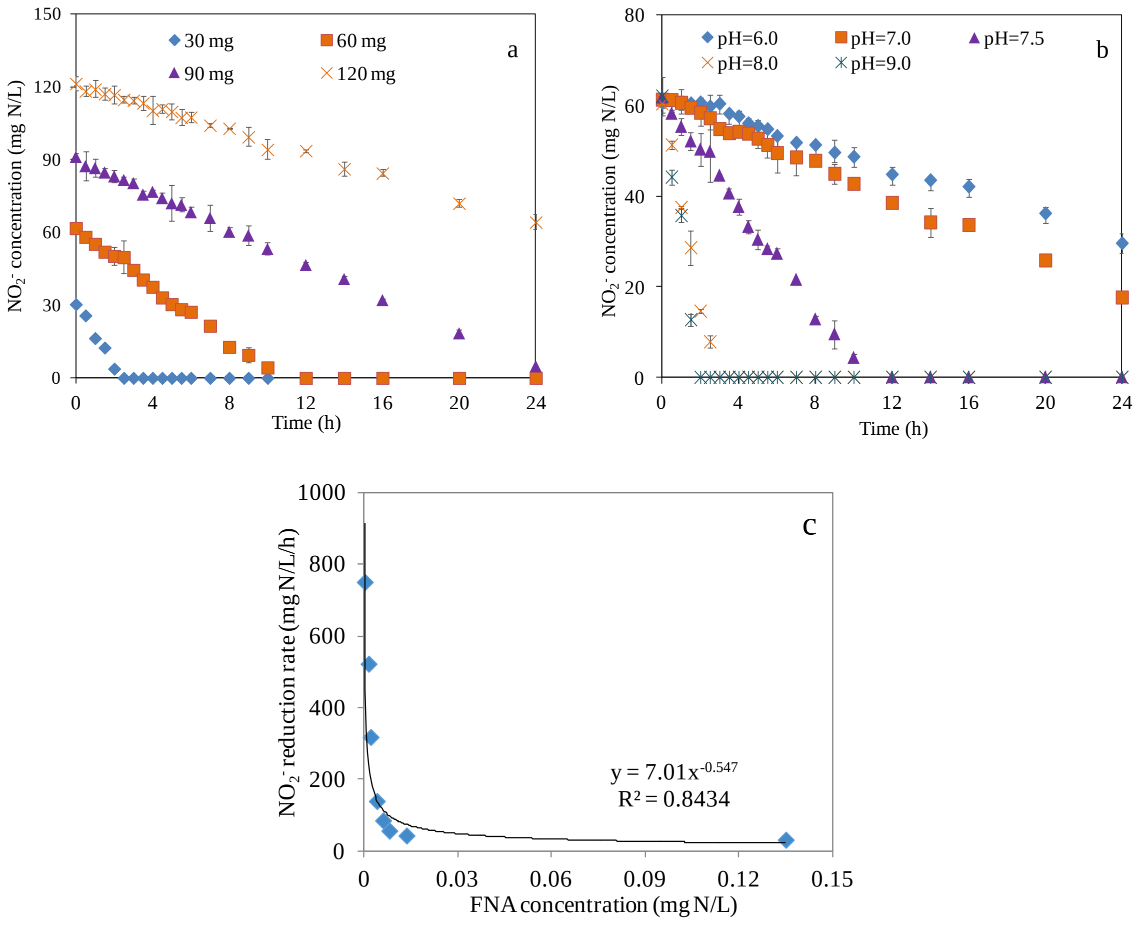

Figure S1. Batch Test II results - biomass-specific denitritation activities under different initial NO_2^- concentrations, pH and FNA concentrations: (a) Profile of nitrite for Batch Reactors 1 to 4 (under different initial NO_2^- concentrations) in Batch Test II; (b) Profile of nitrite for Batch Reactors 5 to 8 (under different pH) in Batch Test II; (c) relationship between NO_2^- reduction rates and initial FNA concentrations in each reactor of Batch Test II.
